# Neutrophil-to-lymphocyte ratio may be associated with the outcome in patients with prostate cancer

**DOI:** 10.1186/s40064-015-1036-1

**Published:** 2015-06-12

**Authors:** Daniele Minardi, M Scartozzi, L Montesi, M Santoni, L Burattini, M Bianconi, V Lacetera, G Milanese, S Cascinu, G Muzzonigro

**Affiliations:** Dipartimento di Scienze Cliniche e Specialistiche, Sezione di Urologia, Università Politecnica delle Marche, Azienda Ospedaliero-Universitaria Ospedali Riuniti, Ancona, Italy; Dipartimento di Oncologia Medica, Università Politecnica delle Marche, Azienda Ospedaliero-Universitaria Ospedali Riuniti, Ancona, Italy; Clinica Urologica, Università Politecnica delle Marche, A.O. Ospedali Riuniti, Via Conca 71, 60020 Ancona, Italy

**Keywords:** Prostate cancer, Neutrophil-to-lymphocyte ratio, Progression free survival

## Abstract

**Purpose:**

Evidences have shown that neutrophil-to-lymphocyte ratio (NLR) has a prognostic value in patients with cancer. We wanted to test the prognostic significance of NLR in prostatic cancer of patients who are candidate to radical prostatectomy.

**Methods:**

We have considered 731 patients. Complete demographic data including age, tumor stage, Gleason score, complete blood count and serum biochemical profile were collected. Pre-treatment percentage of neutrophils and NLR were considered, and correlated with patients data and recurrence free survival.

**Results:**

389 patients were evaluated, mean age 65 years, mean follow-up 51.5 months, mean recurrence free survival 51.3 months. Total neutrophil count does not correlate with biochemical recurrence and disease free survival. Patients with a value higher of 60% of neutrophils are more likely to have a recurrence. Patients with a total lymphocyte count <1,500 have a higher rate of relapse. NLR was not correlated with baseline total PSA, with Gleason score and with pathological stage; patients with a NLR >3 has a higher incidence of recurrence. In multivariate analysis including age, total PSA and NLR, NLR is the most important factor able to predict recurrence. There are some limitations to this study; first, this is a retrospective study, and the total number of patients analyzed is relatively small.

**Conclusions:**

Our study suggests that pre-treatment NLR may be associated with disease free survival in patients with prostate cancer, and could be introduced in clinical practice. NLR has the advantage of low economic cost and wide availability.

**Electronic supplementary material:**

The online version of this article (doi:10.1186/s40064-015-1036-1) contains supplementary material, which is available to authorized users.

## Background

Inflammation is a critical component in the pathogenesis of cancer. It is also important in the progression of cancer (Pinato et al. 2014).

The excess of pro-inflammatory cytokines in the organism of a cancer patient commonly leads to an acute phase reaction that reflects both the disease activity as well as the host’s innate response towards the tumor. So far, the prognostic significance of systemic inflammatory reaction in solid tumors as a potential marker of clinical outcome, has been relatively ignored. However, the relationship between inflammation and cancer has been explored extensively both in animal models and clinical trials in the past years (Li et al. 2014).

In cancer patients lymphopenia is the surrogate of an impaired cell-mediate immunity, while neutrophilia is acknowledged as a response to systematic inflammation (Grivennikov et al. 2010). Neutrophil-to-lymphocyte ratio (NLR) is suggested as a marker for general immune response to various stress stimuli. NLR was described to be correlated with the severity of clinical progress in severely ill patients in the intensive care unit (Zahorec 2001); emerging evidences have shown that NLR has a prognostic value in patients with solid tumors (McMillan 2009); pretreatment high neutrophil count has been reported as a poor prognostic factor for survival in patients with renal cell carcinoma (Négrier et al. 2002), metastatic melanoma (Schmidt et al. 2005), and advanced non-small-cell lung cancer (Teramukai et al. 2009); in addition to be a prognostic score in patients with cancer (Proctor et al. 2011; Wei et al. 2014; Rossi et al. 2015; Pinato et al. 2012), NLR was found to be an helpful tool in the prediction of response to treatment (Edge et al. 2010).

The aim of this study is therefore to test the prognostic significance of the pre-operative NLR in prostatic cancer of patients who are candidate to radical prostatectomy (RP) and to evaluate whether this parameter provides additional prognostic information to well-established clinic and pathological parameters, in particular predicting the biochemical recurrence and disease free survival.

## Methods

We retrospectively analyzed patients that underwent radical prostatectomy registered in a large database. Among 731 patients operated at our Institution between 2005 and 2011 for clinically localized prostate cancer, we considered only patients who did not receive neo-adjuvant therapy or did not undergo adjuvant therapy (both hormones or radiation); also patients with factors that may influence the NLR such as steroids, chronic lymphatic leukemia, infection or other acute events during the month before the NLR measurements were excluded.

All the patients underwent open radical prostatectomy and extended pelvic lymph nodes dissection, including external, internal and obturatory lymph nodes.

Complete demographic data including age, tumor stage, Gleason score at biopsy and at final pathological examination, surgical margin status (positive vs negative) were collected, together with the complete blood count and serum biochemical profile. All the clinic and pathologic data were retrieved from the database.

TNM classification was according to 2010 TNM system (Pinato et al. 2012). The laboratory data, including neutrophil and lymphocyte count, were obtained from before the prostate biopsy and 1 day before surgical intervention.

Neutrophil-to-lymphocyte ratio was calculated by dividing the absolute neutrophil count by the absolute lymphocyte count. Potential factors associated with outcome were evaluated including age, total PSA, Gleason score in the RP specimen, pathological stage, neutrophilia and NLR. Pre-treatment percentage of neutrophils and NLR were considered. The value of NLR that best discriminated between good and poor outcome, which is the most significant *p* value according to the long-rank test, was determined by testing all possible cutoffs.

Follow-up visits after a radical prostatectomy were usually scheduled shortly after surgery, every 3 months for 2 years, then every 6 months up to 5 years, and annually thereafter; follow-up examination included digital rectal examination (DRE) and PSA; radiological investigations (abdominal or thoracic CT and/or RMN and/or PET, Positron Emission Tomography, scan and/or bone scan) were performed in case of rising PSA (Moul 2000) or when clinically required; disease recurrence was defined as evidence of measurable disease on imaging, including CT, MR imaging, PET scan, bone scan or ultrasound, and cytological/histological evaluation of suspected lesions.

### Statistical analysis

Pearson Chi-square test was used to assess any association between categorical variables. Univariate analysis of the different clinical factors associated with survival was carried out using Kaplan–Meier statistics and log-rank test. Each factor was tested for its independent prognostic value using multivariate analysis according to Cox proportional hazard model using SPSS statistical package version 19 (IBM SPSS Inc., USA).

### Ethics statement

All the patients included in this study had given explicit written consent for their information to be stored in the hospital database and used for research. All clinical investigations were conducted according to the principles expressed in the Declaration of Helsinki. The Institutional Review Board of the Azienda Ospedaliero-Universitaria Ospedali Riuniti of Ancona approved the study design.

## Results

Of the 731 patients, 389 patients met the inclusion criteria and were considered for the study purpose and included in the NLR analysis; mean patients age was 65 years (range 42–77), with a mean follow-up time of 51.5 months (range 9–108). Mean recurrence free survival was 51.3 months (range 9–108) (Additional file 1: Table S1).

We have found a statistically significant correlation (p < 0.01) between Gleason score and biochemical failure.

Median neutrophil count was 3,670 mm^3^, median lymphocyte count was 1,510 mm^3^ and median NLR was 2.4.

**Figure 1:**
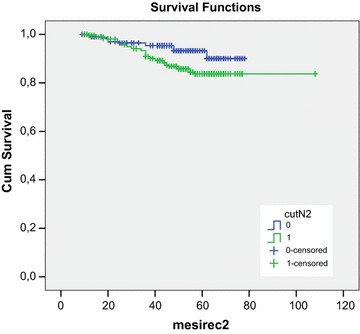
Recurrence free survival in patient with percentage of neutrophils lower than 60% (0) and higher than 60% (1).

We have considered the total neutrophil count and we have found that it has not a statistically significant correlation with biochemical recurrence and disease free survival. Considering then the percentage of neutrophils in the leukocyte formula, we have observed that a cut-off value of 60% is able to discriminate patients; in fact those having an higher value are more likely to have a recurrence (p < 0.05) than those with a lower value (Figure 1); by linear regression analysis however this parameter is less powerful than baseline PSA and Gleason score to predict recurrence.

**Figure 2:**
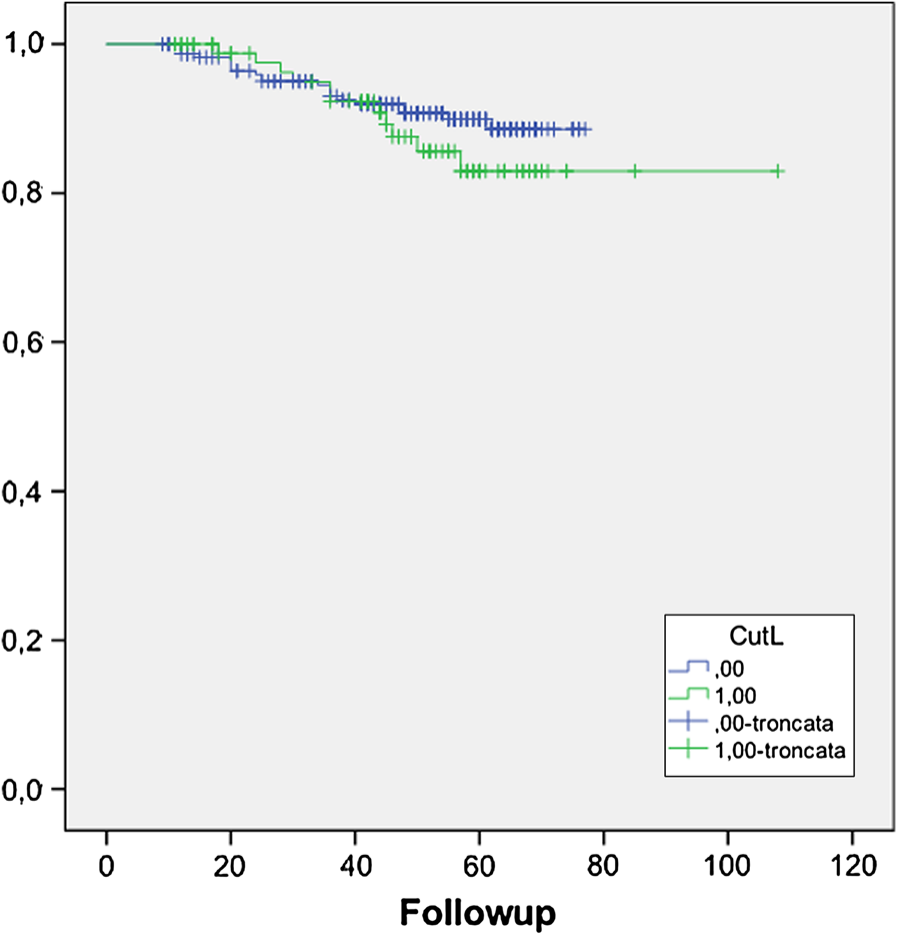
Recurrence free survival in patient with total lymphocyte count >1,500 mm^3^ (0) and <1,500 mm^3^ (1).

We have then considered total lymphocyte count, and we have found that patients with <1,500 have a higher rate of relapse, compared to those with >1,500 mm^3^ (Figure 2); we were not able to find a cut-off value of lymphocyte percentage of the leukocyte formula able to predict recurrence.

Finally we have considered NLR ratio; when NLR was analyzed as a dichotomous variable, a cut-point of three provided the strongest prognostic value in our data set, therefore this level was chosen for further studies.

**Figure 3:**
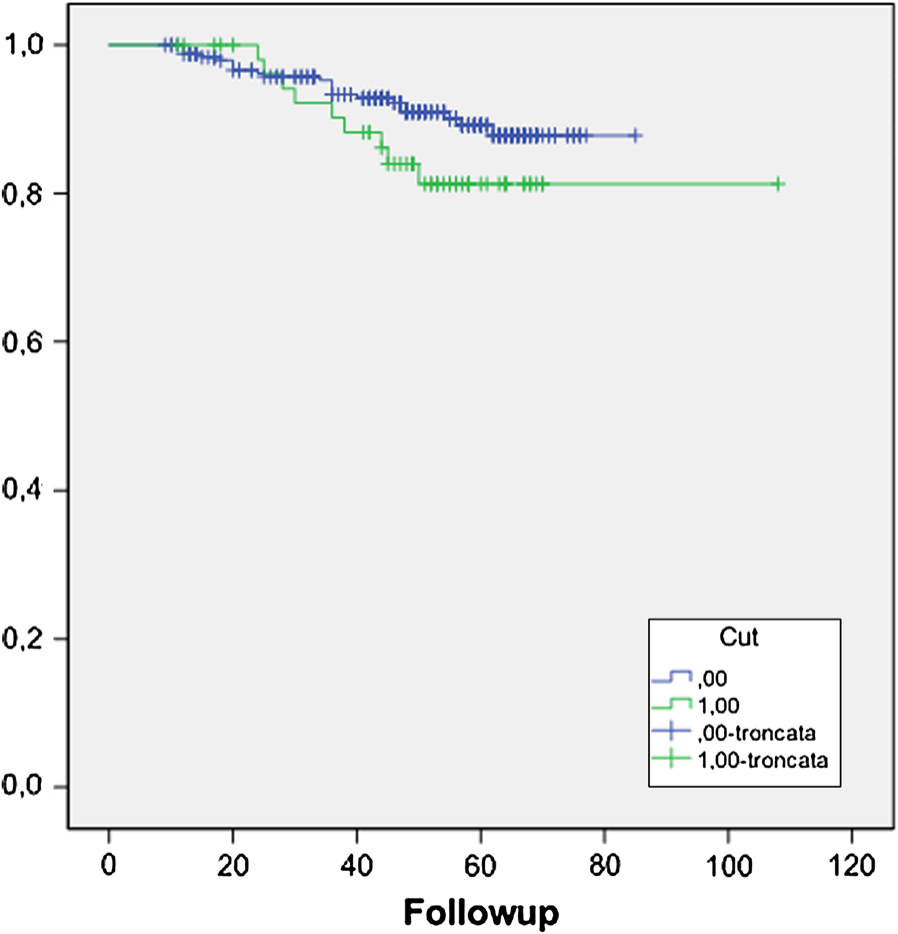
Recurrence free survival in patients with NLR <3 (0) and >3 (1).

NLR was not correlated with baseline total PSA, with Gleason score and with pathological stage; but we have found that patients with a NLR >3 has a higher incidence of recurrence than patients with a NLR <3 (Figure 3).

Multivariate analysis was conducted, primarily, on three factors that can be easily collected at the first consultation in the outpatient clinic of patients with prostate cancer: age, total PSA and NLR; NLR is the most important factor able to predict recurrence (p < 0.05) (Additional file 2: Table S2).

In the multivariate analysis, considering total neutrophil and lymphocyte count, NLR, age, total PSA, Gleason grading, stage, and surgical margin status, the latter two parameters resulted to be the most important factor predictive of recurrence (Additional file 3: Table S3).

## Discussion

Prostate cancer is a common malignancy and a leading cause of cancer-related death. Although the long natural history of this disease usually allows several levels of therapeutic intervention, most patients who have recurrent disease following initial definitive treatment (~1/3 of all patients diagnosed) will ultimately develop castrate-resistant disease, the lethal form of the disease.

Active immunotherapy or anti-cancer vaccination are treatment approaches currently investigated, a strategy designed to elicit and/or augment anti-tumor immune responses. Prostate cancer has proven to be particularly sensible to immunotherapeutic intervention, given the possibility of antigen loss variants as a means for tumors to evade immune detection (Olson et al. 2013).

There is strong linkage between inflammation and cancer; cancer related inflammation reduces antitumor immunity by recruiting regulatory T cells and activating chemokines, which results in tumor growth and metastasis. The mechanism between cancer, neutrophilia and leukocytosis remains unclear; however, cancer has been shown to produce granulocyte colony-stimulating factors (Feng et al. 2013). An enhanced neutrophil response and/or suppression of lymphocytes leading to a high NLR might promote carcinogenesis and inhibit antitumor immune response. Molecular signaling and inflammatory mediators triggered pathways could promote cancer cell proliferation, angiogenesis and metastasis (Gueron et al. 2012). Inflammatory cells have powerful effects on tumor development. Early in the neoplastic process, these cells produce an attractive environment for tumor growth, facilitating genomic instability and promoting angiogenesis; but inflammatory response is anti-tumor (Coussens and Werb 2002).

Nowadays, tumor stage and other clinical parameters such as PSA and Gleason score are used to obtain prognostic information and could be helpful choosing appropriate treatment strategies for patients with prostate cancer. Peripheral blood tests before treatment or at the time of diagnosis may reflect inflammatory conditions within the tumor. NLR calculated from a convenient and cheap test could provide appropriate prognostic information for the patients in the treatment of prostate cancer (Wei et al. 2014).

The present study suggests that pre-treatment NLR may be associated with progression free survival in patients with prostate cancer.

Patients with neutrophil percentage higher than 60%, with total lymphocyte count lower than 1,500 mm^3^ and with NLR higher than three showed a higher incidence of recurrence; these observations, together with age and total PSA, are able to predict recurrence in our patient population. These markers have the advantage of low economic cost and wide availability.

Patients with pre-treatment NLR <3 had a longer progression free survival; our observation suggests that exploring the combined impact of neutrophil and lymphocyte counts may provide prognostic informations.

The association between NLR and outcome is complex and remains to be elucidated. A high NLR reflects both a heightened neutrophil-dependent inflammatory reaction and a decreased lymphocyte mediated anti-tumor immune response, that contribute to aggressive tumor biology, cancer progression and poor prognosis (Keizman et al. 2012; Ohno et al. 2010; An et al. 2010). It has been observed that the neutrophil count alone may not reflect the prognostic informations of a decreased lymphocyte mediated immune response, and a low lymphocyte count alone may not reflect the neutrophil driven tumorigenesis process (18); thus the NLR may reflect the combined prognostic information of these processes, and be a stronger predictor of anti-tumor immune response and patients outcome.

There are some limitations to this study; first, this is a retrospective study, which is susceptible to bias in data selection and analysis; the total number of patients analyzed is relatively small and included patients who were treated by radical prostatectomy. It was not possible to include in this study patients who were not treated by surgery, but by hormones or radiotherapy, because data would have been altered; furthermore, NLR differs among individuals and can be influenced by concurrent infection and drugs.

Despite these limitations, our study suggests that pre-treatment NLR may be associated with disease free survival in patients with prostate cancer, and could be introduced in clinical practice. We have also found that NLR associated with baseline PSA is a strong predictor of recurrence free survival and should be taken into consideration when planning treatment for prostate cancer, in particular adjuvant therapy after RP or more aggressive therapy in patients who are not candidates for surgery. NLR has the advantage of low economic cost and wide availability. The results of our study encourage routinely monitoring of NLR to predict recurrence in prostate cancer patients.

## Additional files

Additional file 1:
**Table S1.** Patients characteristics. Additional file 2:
**Table S2.** Age, total PSA and NLR as predictor of recurrence. Additional file 3:
** Table S3.** Multivariate analysis showing that stage and margin status are the most important factors in predicting recurrence.

## References

[CR1] An X, Ding PR, Li YH, Wang FH, Shi YX, Wang ZQ (2010). Elevated neutrophil to lymphocyte ratio predicts survival in advanced pancreatic cancer. Biomarkers.

[CR2] Coussens LM, Werb Z (2002). Inflammation and cancer. Nature.

[CR3] Edge S, Byrd DR, Compton CC, Fritz AG, Greene FL, Trotti A (2010). American joint committee on cancer staging manual.

[CR4] Feng JF, Huang Y, Liu JS (2013). Combination of neutrophil lymphocyte ratio and platelet lymphocyte ration is a useful predictor of postoperative survival in patients with esophageal squamous cell carcinoma. Onco Targets Therapy.

[CR5] Grivennikov SI, Greten FR, Karin M (2010). Immunity, inflammation and cancer. Cell.

[CR6] Gueron G, De Servi A, Vazquez E (2012). Advanced prostate cancer: reinforcing the strings between inflammation and the metastatic behavior. Prostate Cancer Prostatic Dis.

[CR7] Keizman D, Ish-Shalom M, Huang P, Eisenberg MA, Pili R, Hammers H (2012). The association of pre-treatment neutrophil to lymphocyte ratio with response rate, progression free survival, and overall survival of patients treated with sunitinib for metastatic renal cell carcinoma. Eur J Cancer.

[CR8] Li MX, Liu XM, Zhang XF, Zhang JF, Wang WL, Zhu Y (2014). Prognostic role of neutrophil-to-lymphocyte ratio in colorectal cancer: a systematic review. Int J Cancer.

[CR9] McMillan DC (2009). Systemic inflammation, nutritional status and survival in patients with cancer. Curr Opin Clin Nutr Metab Care.

[CR10] Moul JW (2000). Prostate specific antigen only progression of prostate cancer. J Urol.

[CR11] Négrier S, Escudier B, Gomez F, Douillard J-Y, Ravaud A, Chevreau C (2002). Prognostic factors of survival and rapid progression in 782 patients with metastatic renal carcinoma treated by cytokines: a report from the Group Français d’Immunothérapie. Ann Oncol.

[CR12] Ohno Y, Nakashima J, Ohori M, Hatano T, Tachibana M (2010). Pretreatment neutrophil-to-lymphocyte ratio as an independent predictor of recurrence in patients with nonmetastatic renal cell carcinoma. J Urol.

[CR13] Olson BM, Johnson LE, McNeel DG (2013). The androgen receptor: a biologically relevant vaccine target for the treatment of prostate cancer. Cancer Immunol Immunother.

[CR14] Pinato DJ, Stebbing J, Ishizuka M, Khan SA, Wasan HS, North BV (2012). A novel and validated prognostic index in hepatocellular carcinoma: the inflammation based index (IBI). J Hepatol.

[CR15] Pinato DJ, Stavraka C, Flynn MJ, Forster MD, O’Cathail SM, Seckl MJ (2014). An inflammation-based score can optimize the selection of patients with advanced cancer considered for early phase clinical trial. Plos One.

[CR16] Proctor MJ, Morrison DS, Talwar D, Balmer SM, Fletcher CD, O’Reilly DS (2011). A comparison of inflammation-based prognostic scores in patients with cancer. A Glasgow inflammation Outcome Study. Eur J Cancer.

[CR17] Rossi L, Santoni M, Crabb SJ, Scarpi E, Burattini L, Chau C (2015). High neutrophil-to-lymphocyte ratio persistent during first-line chemotherapy predicts poor clinical outcome in patients with advanced urothelial cancer. Ann Surg Oncol.

[CR18] Schmidt H, Bastholt L, Geertsen P, Douillard JY, Ravaud A, Chevreau C (2005). Elevated neutrophil and monocyte counts in peripheral blood are associated with poor survival in patients with metastatic melanoma: a prognostic model. Br J Cancer.

[CR19] Teramukai S, Kitano T, Kishida Y, Kawahara M, Kubota K, Komuta K (2009). Pretreatment neutrophil count as an independent prognostic factor in advanced non-small-cell lung cancer: an analysis of Japan Multinational Trial Organization LC00-03. Eur J Cancer.

[CR20] Wei Y, Jiang YZ, Qian WH (2014). Prognostic role of NLR in urinary cancers: a meta-analysis. Plos One.

[CR21] Zahorec R (2001). Ratio of neutrophil to lymphocyte count—rapid and simple parameter of systemic inflammation and stress in critically ill. Bratisl Lek Listy.

