# Supplementary material for: Neutrophil-to-lymphocyte ratio may be associated with the outcome in patients with prostate cancer
Source: Springerplus. 2015 Jun 12;4:255. doi: 10.1186/s40064-015-1036-1 (PMC4463949; doi:10.1186/s40064-015-1036-1)
Supplement: Additional file 1: — Table S1. Patients characteristics. [file 40064_2015_1036_MOESM1_ESM.doc]

Table S1 Patients characteristics

| Patients | 389 | |
| --- | --- | --- |
| Age | 65 | |
| Range | 42-77 | |
| Stage | *n. pts*  33  10  123  61  169  4 | *stage*  pt2a  pT2b  pT3a  pT3b  pT3c  pT4 |
| Grade | *n. pts*  152  140  57  16      4 | *grade*  3+3=6  3+4=7  4+3=7  4+4=8  5+4=9  4+5=9  5+5=10 |
| Median neutrophil count | 3670 mm^3^ | |
| Median lymphocyte count | 1510 mm^3^ | |
| NLR | 2.4 | |
