# Supplementary material for: Neutrophil-to-lymphocyte ratio may be associated with the outcome in patients with prostate cancer
Source: Springerplus. 2015 Jun 12;4:255. doi: 10.1186/s40064-015-1036-1 (PMC4463949; doi:10.1186/s40064-015-1036-1)
Supplement: Additional file 2: — Table S2. Age, total PSA and NLR as predictor of recurrence. [file 40064_2015_1036_MOESM2_ESM.doc]

Table S2 Age, total PSA and NLR as predictor of recurrence

|  | | | | | | |
| --- | --- | --- | --- | --- | --- | --- |
|  | B | SE | Wald | df | Sig. | Exp(B) |
| Age | -,037 | ,028 | 1,667 | 1 | ,197 | ,964 |
| NLR | ,329 | ,150 | 4,831 | 1 | ,028 | 1,390 |
| PSA | ,002 | ,006 | ,106 | 1 | ,745 | 1,002 |
