# Supplementary material for: Neutrophil-to-lymphocyte ratio may be associated with the outcome in patients with prostate cancer
Source: Springerplus. 2015 Jun 12;4:255. doi: 10.1186/s40064-015-1036-1 (PMC4463949; doi:10.1186/s40064-015-1036-1)
Supplement: Additional file 3: — Table S3. Multivariate analysis showing that stage and margin status are the most important factors in predicting recurrence. [file 40064_2015_1036_MOESM3_ESM.doc]

Table S3 Multivariate analysis showing that stage and margin status are the most important factors in predicting recurrence

|  | | | | | | |
| --- | --- | --- | --- | --- | --- | --- |
|  | B | SE | Wald | df | Sig. | Exp(B) |
| L | ,001 | ,001 | 1,314 | 1 | ,252 | 1,001 |
| Age | -,023 | ,045 | ,269 | 1 | ,604 | ,977 |
| GleasonScore | ,276 | ,204 | 1,829 | 1 | ,176 | 1,318 |
| Stage | ,622 | ,310 | 4,040 | 1 | ,044 | 1,864 |
| **Margins** | **,610** | **,301** | **4,001** | **1** | **,037** | **1,784** |
| N | ,000 | ,000 | ,652 | 1 | ,419 | 1,000 |
| NLR | ,624 | ,516 | 1,461 | 1 | ,227 | 1,866 |
| PSA | -,001 | ,015 | ,005 | 1 | ,944 | ,999 |
